# Ectromelia virus suppresses expression of cathepsins and cystatins in conventional dendritic cells to efficiently execute the replication process

**DOI:** 10.1186/s12866-019-1471-1

**Published:** 2019-05-10

**Authors:** Magdalena Bossowska-Nowicka, Matylda B. Mielcarska, Marta Romaniewicz, Monika M. Kaczmarek, Karolina P. Gregorczyk-Zboroch, Justyna Struzik, Marta Grodzik, Małgorzata M. Gieryńska, Felix N. Toka, Lidia Szulc-Dąbrowska

**Affiliations:** 1 Division of Immunology, Department of Preclinical Sciences, Faculty of Veterinary Medicine, Warsaw University of Life Sciences – SGGW, Ciszewskiego 8, 02-786 Warsaw, Poland; 2 Molecular Biology Laboratory, Institute of Animal Reproduction and Food Research, Polish Academy of Sciences, Olsztyn, Poland; 3 Division of Nanobiotechnology, Department of Animal Nutrition and Biotechnology, Faculty of Animal Sciences, Warsaw University of Life Sciences-SGGW, Warsaw, Poland; 4 Center for Integrative Mammalian Research, Ross University School of Veterinary Medicine, Basseterre, St. Kitts and Nevis

**Keywords:** Cathepsins, Cystatins, Dendritic cells, ECTV

## Abstract

**Background:**

Cathepsins are a group of endosomal proteases present in many cells including dendritic cells (DCs). The activity of cathepsins is regulated by their endogenous inhibitors – cystatins. Cathepsins are crucial to antigen processing during viral and bacterial infections, and as such are a prerequisite to antigen presentation in the context of major histocompatibility complex class I and II molecules. Due to the involvement of DCs in both innate and adaptive immune responses, and the quest to understand the impact of poxvirus infection on host cells, we investigated the influence of ectromelia virus (ECTV) infection on cathepsin and cystatin levels in murine conventional DCs (cDCs). ECTV is a poxvirus that has evolved many mechanisms to avoid host immune response and is able to replicate productively in DCs.

**Results:**

Our results showed that ECTV-infection of JAWS II DCs and primary murine GM-CSF-derived bone marrow cells down-regulated both mRNA and protein of cathepsin B, L and S, and cystatin B and C, particularly during the later stages of infection. Moreover, the activity of cathepsin B, L and S was confirmed to be diminished especially at later stages of infection in JAWS II cells. Consequently, ECTV-infected DCs had diminished ability to endocytose and process a soluble antigen. Close examination of cellular protein distribution showed that beginning from early stages of infection, the remnants of cathepsin L and cystatin B co-localized and partially co-localized with viral replication centers (viral factories), respectively. Moreover, viral yield increased in cDCs treated with siRNA against cathepsin B, L or S and subsequently infected with ECTV.

**Conclusions:**

Taken together, our results indicate that infection of cDCs with ECTV suppresses cathepsins and cystatins, and alters their cellular distribution which impairs the cDC function. We propose this as an additional viral strategy to escape immune responses, enabling the virus to replicate effectively in infected cells.

**Electronic supplementary material:**

The online version of this article (10.1186/s12866-019-1471-1) contains supplementary material, which is available to authorized users.

## Background

Dendritic cells (DCs) are professional antigen-presenting cells (APCs) found in all epithelial tissues and are strategically located at the sites of pathogen entry. A number of studies have established that DCs are an important link between innate and adaptive immunity because of their capability to innately detect pathogens through receptors such as Toll-like receptors, (TLRs), and also the ability to process and present antigens to lymphocytes [1, 2]. Adaptive immune responses are initiated by CD4^+^ or CD8^+^ T lymphocytes recognition of antigens associated with major histocompatibility complex (MHC) class II or class I molecules, respectively, displayed on DCs surface. Therefore, processing of antigens, which occurs in phagosomes/endosomes or the proteasome is an important step before epitope association with MHC molecules [3].

The major group of enzymes responsible for the degradation of exogenous or endogenous antigens is mostly the protease. Among them, the best known and well characterized are cysteine cathepsins. Cathepsins are essential enzymes involved in protein degradation, and extracellular matrix remodeling in all cell types and tissues [4]. They are produced as inactive enzymes (zymogenes) within the lysosomes. Cathepsins undergo activation by autocatalysis due to low pH or they are activated by other proteases, such as pepsin and cathepsin D [5]. Cysteine cathepsins are present and active in lysosomes, nucleus, cytoplasm and cell membrane [6]. Furthermore, cathepsins are also present in the extracellular space and secretory vesicles of some cells, not only as zymogens, but also as active proteases [7].

It appears that besides the role of cysteine cathepsins in antigen processing, they also participate in cell entry of many types of viruses, including Ebola virus (EBOV) [8, 9], human immunodeficiency virus (HIV) [10, 11] and reoviruses [12, 13]. However, the exact role of cathepsins during poxvirus infection has not yet been adequately investigated.

The mousepox virus (ectromelia virus, ECTV) is a double stranded DNA virus that belongs to *Poxviridae* family of viruses in the genus *Orthopoxvirus,* the same genus that encompasses variola virus (VARV, the causative agent of smallpox) and vaccinia virus (VACV) [14]. Infection of mice with ECTV causes symptoms much similar to those observed in people infected with VARV [15, 16]. Moreover, infection with ECTV is characterized, in contrast to VACV, by a narrow host range and the course of infection is very similar to that observed in humans infected with VARV [17]. Orthopoxviruses have well-evolved immune evasion strategies to elude and manipulate the host defense mechanisms [18–20]. The activity of cysteine cathepsins is regulated by endogenous protein inhibitors called cystatins. There are three types of cystatins: type 1 cystatins, also called stefins (A and B) are generally located intracellularly; type 2 cystatins: C, D, E/M, F, S, SN and SA are extracellular proteins, and type 3 cystatins – kininogens which are multifunctional plasma proteins [21, 22]. Beyond regulation of cysteine cathepsins activity cystatins also protect host cells from pathogens that use cysteine proteases for cell entry [23].

Our previous studies showed that mRNA and protein level of cathepsin B, L and S was downregulated during infection of murine peritoneal macrophages with the mousepox virus. Furthermore, siRNA knockdown of cathepsin B and L in macrophage cell line RAW 264.7 resulted in increased viral yield in supernatants collected 48 h post infection (pi) with ECTV [24]. Interestingly, Wang and co-authors [25] indicated that mRNA and protein level of cathepsins B, D and S is reduced in human B cells during VACV infection, which probably alters the MHC class II antigen presentation pathway. However, it is not known if cathepsin expression in conventional DCs (cDCs) is affected during infection with ECTV.

In the present study we asked whether cathepsins are targeted by the replicating ECTV in cDCs in in vitro conditions. For this purpose we used an immortalized immature GM-CSF–dependent DC line JAWS II established from bone marrow cells of p53^−/−^ C57BL/6 mice [26], and primary murine GM-CSF-derived bone marrow (GM-BM) cells (formerly described as bone marrow derived DCs – BMDCs) derived from C57BL/6 mice. The latter cell culture in fact represents a heterogeneous cell population, consisting of DCs and macrophages, what may influence the interpretation of obtained results [27]. JAWS II cells has been shown to be a convenient substitute for primary BMDCs, however, they may harbor inherent differences resulted from the immortalization process and/or from the p53-deficiency [26]. Having the above considerations in mind, using both cell cultures minimize the risk of misinterpretation or over-interpretation of obtained results. GM-CSF-derived bone marrow cells from in vitro cultures phenotypically and functionally resemble monocyte-derived inflammatory DCs and non-lymphoid tissue DCs, such as interstitial DCs found in vivo in the mouse [28, 29].

Our results show profound down-regulation of gene and protein expression for cathepsin B, L and S at 24 hpi with ECTV in JAWS II and GM-BM cells. Similarly, cystatin B and C mRNA and protein levels were markedly reduced in infected cells. Additionally, cathepsin L was found to co-localize with viral factories during the entire replication cycle in both type of cells. Moreover, antigen uptake and processing was impaired in infected JAWS II cells at 12 and 24 hpi and in infected GM-BM cells at 24 hpi. Importantly, we show more efficient replication of ECTV in JAWS II and GM-BM cells with the minimal level of cathepsin B, L or S. These results show that ECTV may impair the function and distribution of cathepsins as well as cystatins in cDCs, accompanied by altered capacity to process an exogenous antigen. This may serve as a viral strategy to escape immune response and presumably enables the virus to replicate effectively in infected cells.

## Results

### ECTV induces early apoptosis in JAWS II dendritic cells at later stages of infection

**Fig. 1:**
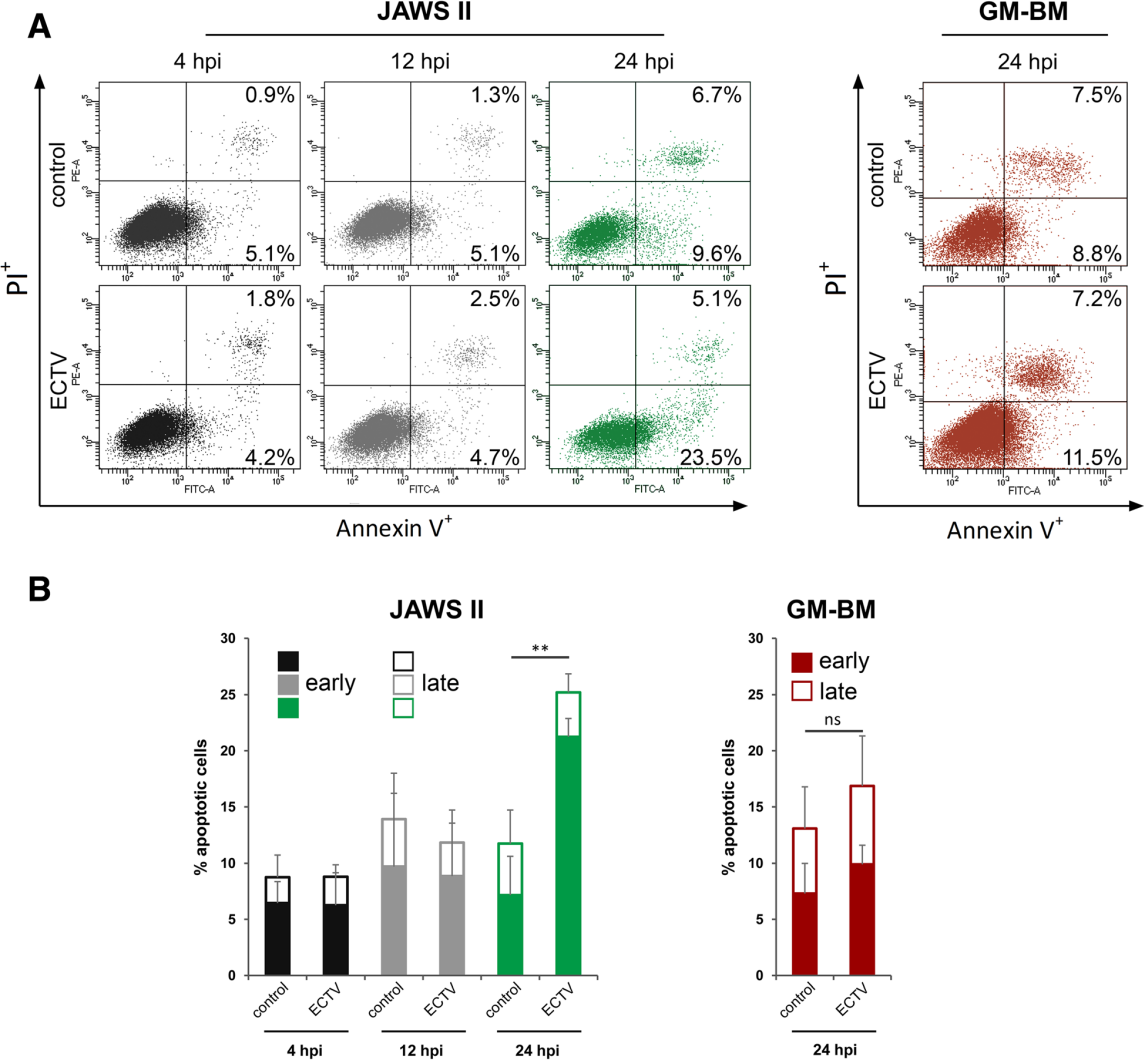
Apoptotic rates in JAWS II and GM-BM cells during ECTV infection. **a** Representative dot plots showing the percentage of early (Annexin V-FITC^+^ and PI^–^) and late (Annexin V-FITC^+^ and PI^+^) apoptotic cells in JAWS II and GM-BM cells infected with ECTV at 4, 12 and/or 24 hpi. **b** The percentage (mean ± SD) of early and late apoptotic cells in JAWS II and GM-BM cells at 4, 12 and/or 24 hpi with ECTV from two independent experiments (**p ≤ 0.01)

ECTV is able to productively infect JAWS II [30] and GM-BM [30, 31] cells. Therefore, in order to eliminate the apoptosis bias in interpreting the gene and protein expression data, we first assessed the apoptotic rate in these cells during the infection with ECTV at multiplicity of infection (MOI) = 0.5. Fig. 1 shows that the percentage of early and late apoptotic cells did not change between ECTV-infected and control JAWS II dendritic cells during the first 12 h of infection. However, at 24 hpi the percentage of early apoptotic cells was significantly (*p* ≤ 0.01) higher in ECTV-infected JAWS II cells compared to uninfected control cells. In our recent study [30] we reported that GM-BM infected with ECTV at MOI = 5 did not show increased apoptosis until 12 hpi, therefore, in the present study (GM-BM infected with ECTV at MOI = 0.5) we evaluated the apoptotic rate only at 24 hpi (Fig. 1). The percentage of early apoptotic cells was slightly, but not significantly (*p* ≥ 0.05) increased in GM-BM infected with ECTV compared to uninfected cells. Taken together, our data indicate that cDCs infected with low doses of ECTV undergo early apoptosis only during the later stages of infection.

### ECTV suppresses cathepsin and cystatin mRNA expression in JAWS II and GM-BM cells

**Fig. 2:**
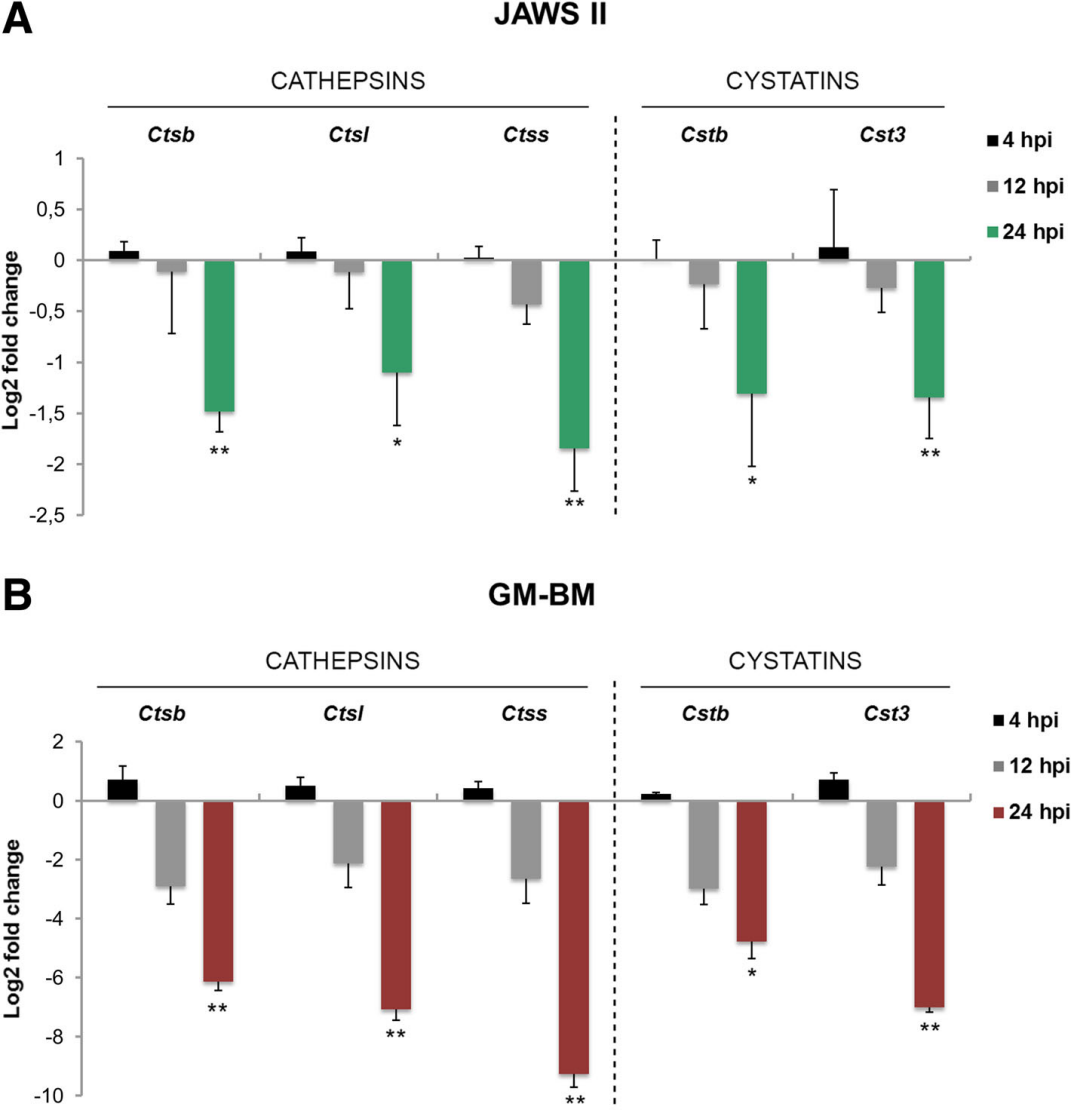
Suppression of cathepsin and cystatin mRNA expression in JAWS II and GM-BM cells during ECTV infection. Log2 fold change of mRNA expression for cathepsins (*Ctsb*, *Ctsl*, *Ctss*) and cystatins (*Cstb*, *Cst3*) in JAWS II (**a**) and GM-BM (**b**) cells following infection with ECTV at 4, 12 and 24 hpi. Data obtained from three independent experiments and presented as mean values of log2 fold change. *p ≤ 0.05; **p ≤ 0.01; ***p ≤ 0.001

Next, we investigated the mRNA expression of cathepsins B, L and S and their inhibitors: cystatins A, B and C during the replication cycle of ECTV in JAWS II and GM-BM cells. The most significant down-regulation of *Ctsb*, *Ctss* (both *p* ≤ 0.01) and *Ctsl* (*p* ≤ 0.05) gene expression was observed at 24 hpi with ECTV in JAWS II cells. Similarly, significant reduction of mRNA expression for *Cstb* (p ≤ 0.05) and *Cst3* (*p* ≤ 0.01) was observed at 24 hpi (Fig. 2a). In contrast, the level of mRNA expression for cystatin A was undetectable in uninfected and infected cells. The gene expression results for cathepsins B, L and S in ECTV-infected GM-BM revealed significant inhibition of *Ctsb* (*p* ≤ 0.01), *Ctsl* (*p* ≤ 0.05) and *Ctss* (*p* ≤ 0.001) expression as early as at 12 hpi (Fig. 2b). Similarly, the reduction of mRNA expression for *Cstb* and *Cst3* at 12 hpi with ECTV was also significant (*p* ≤ 0.001 and *p* ≤ 0.01, respectively) in GM-BM cells. Furthermore, the expression levels of *Ctsb*, *Ctsl*, *Ctss*, *Cstb* and *Cst3* were also decreased at 24 hpi, but the suppression of *Ctss* was the most profound (Fig. 2b). In GM-BM cells the level of mRNA expression for cystatin A was also undetectable in both uninfected and infected cells during the course of ECTV replication. In summary, our data indicate that ECTV negatively regulates the mRNA expression of cathepsins B, L and S, and cystatins B and C in murine cDCs. Such inhibitory effect was evident and occurred earlier during ECTV infection of GM-BM than JAWS II cells.

### Cathepsin and cystatin protein expression in JAWS II and GM-BM cells following infection with ECTV

**Fig. 3:**
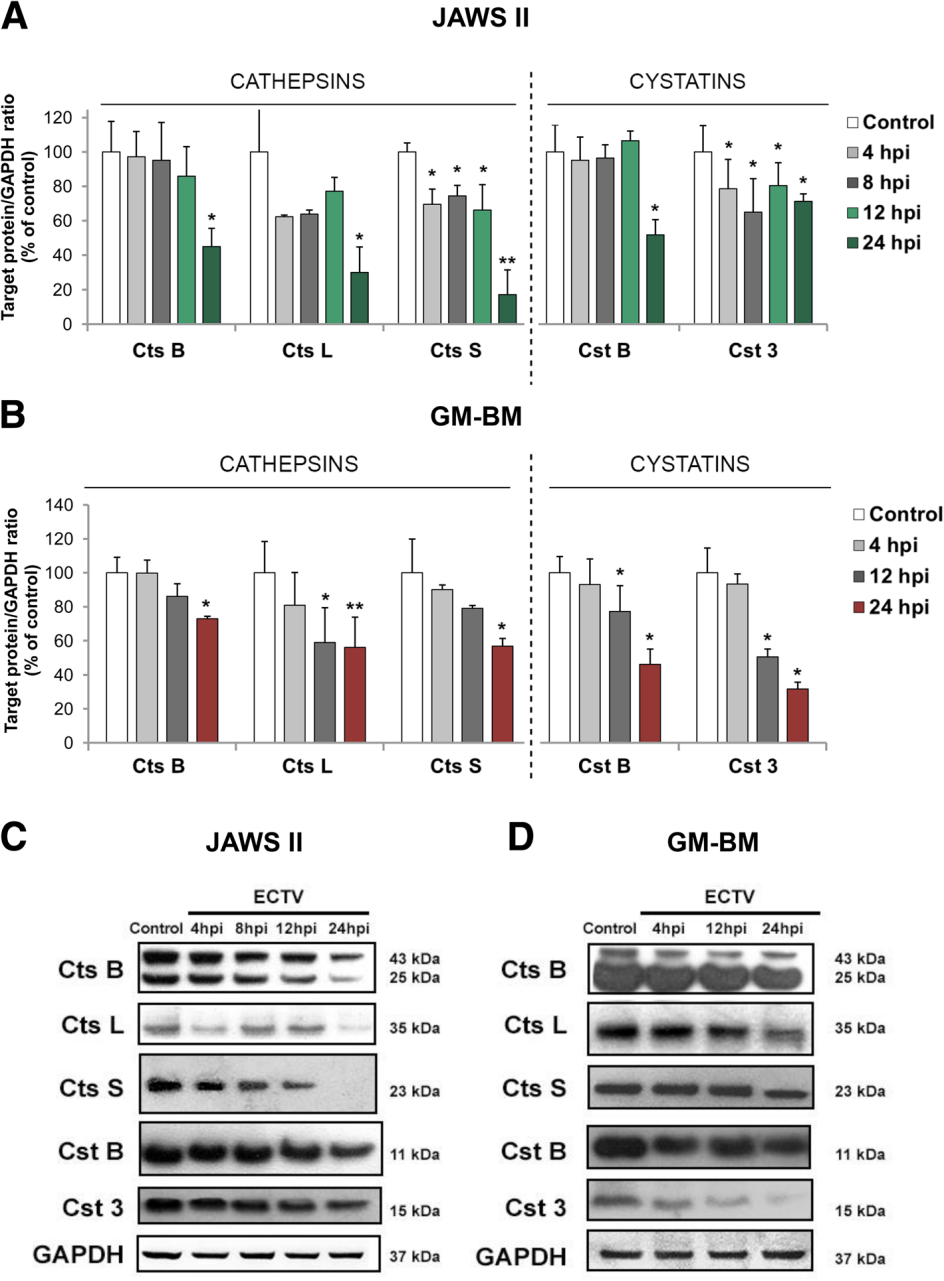
Down-regulation of cathepsin and cystatin protein levels in ECTV-infected JAWS II and GM-BM cells**.** Densitometry analysis of cathepsins (Ctsb, Ctsl and Ctss) and cystatins (Cstb and Cst3) at 4, 8, 12 and/or 24 hpi in JAWS II (**a**) and GM-BM (**b**) cells. The level of each protein was normalized to GAPDH. Data obtained from three independent experiments and presented as the mean ± SD. *p ≤ 0.05; **p ≤ 0.01. Representative Western blots of Ctsb, Ctsl, Ctss, Cstb and Cst3 at 4, 8, 12 and 24 hpi in JAWS II (**c**) and at 4, 12 and 24 hpi in GM-BM cells (**d**)

Because the mRNA expression of cathepsins B, L and S, and cystatins B and C was significantly down-regulated in JAWS II and GM-BM cells following ECTV infection, we focused on assessing the impact of virus infection on the level of protein expression. Reduction in mRNA expression was reflected in the significant down-regulation of Ctss protein at 4, 8 and 12 hpi (*p* ≤ 0.05) with the most inhibition at 24 hpi (*p* ≤ 0.01), and Ctsb and Ctsl (*p* ≤ 0.05) at 24 hpi in JAWS II cells (Fig. 3a). Cystatin B and C levels were also significantly (*p* ≤ 0.05) reduced during ECTV infection, however the reduction of cystatin C was evident at earlier stages of infection (Fig. 3a).

Similar results were observed in GM-BM cells during the course of ECTV infection. Western blot analysis revealed significant down-regulation of cathepsins B and S (*p* ≤ 0.05), cathepsin L (*p* ≤ 0.01), and cystatins B and C (*p* ≤ 0.05) protein expression at 24 hpi, but the levels of cathepsin L, cystatin B and C were significantly (*p* ≤ 0.05) inhibited already at 12 hpi in GM-BM cells (Fig. 3b). The down-regulation of cathepsin and cystatin protein expression was associated with reduced expression of these enzymes at the gene level and is in agreement with our previous results concerning expression of Ctsb, Ctsl and Ctss in peritoneal macrophages isolated from BALB/c and C57BL/6 mice [24]. It is highly likely that ECTV diminishes the expression levels of cathepsins and cystatins in order to perturb immune responses orchestrated by cDCs.

### ECTV infection impairs cathepsin activity in JAWS II cells

**Fig. 4:**
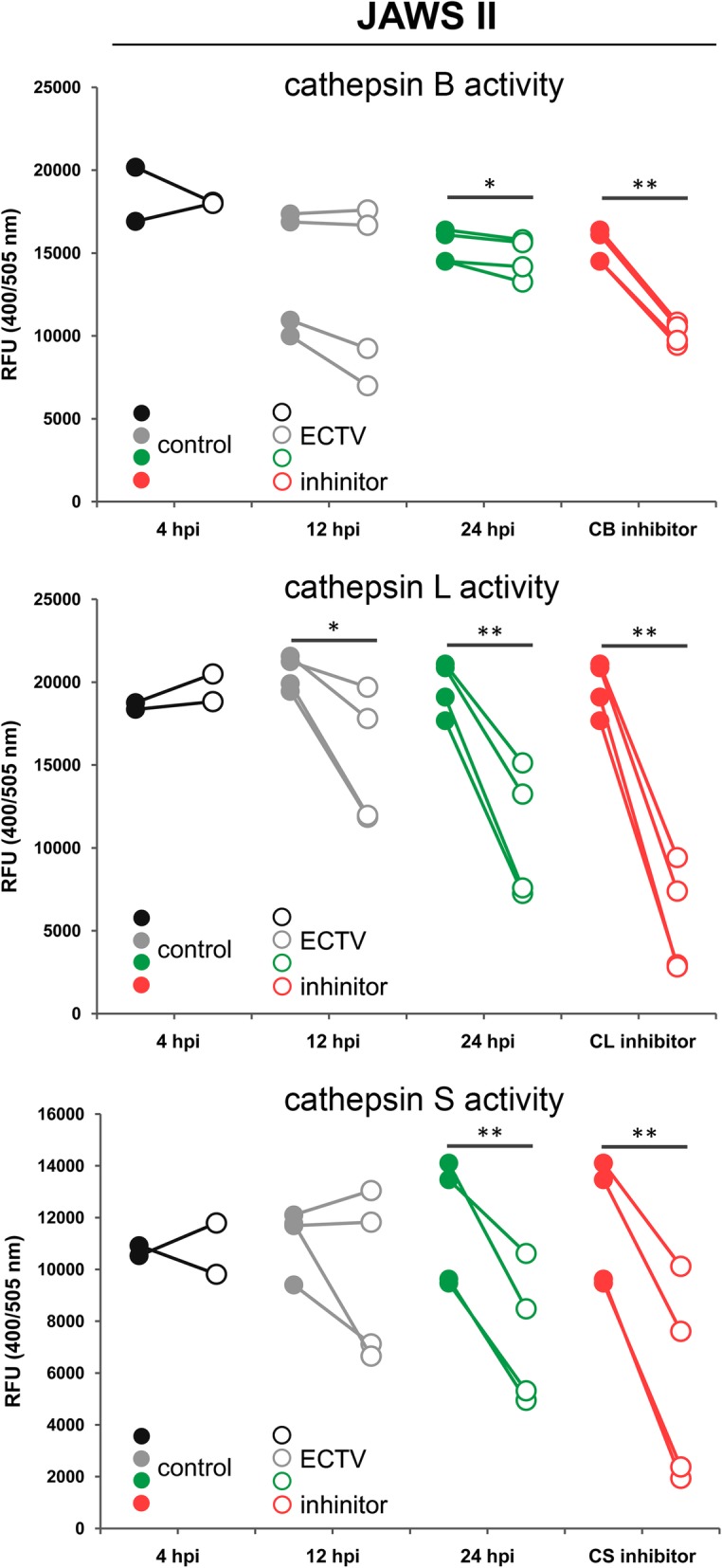
ECTV infection suppresses the activity of cathepsins in JAWS II cells**.** Graphs represent individual data of relative fluorescence units (RFU) from two (4 hpi) or four (12 and 24 hpi) independent experiments (*p ≤ 0.05, **p ≤ 0.01)

Because ECTV infection down-regulates the expression of both cathpesins and their endogenous inhibitors cystatins, we further asked if a change occurs in the activity of cathepsin proteases following infection. To this extent, we analyzed the activity of cathepsins in cell lysates of JAWS II DCs during ECTV replication cycle. At early stages (4 hpi) of infection the activity of all analyzed cathepsins did not change compared to uninfected cells. At 12 hpi the activity of cathepsin L was significantly (*p* ≤ 0.05) decreased, whereas the activity of cathpesins B and S remained unchanged compared to the control cells (Fig. 4). However, at 24 hpi with ECTV the activity of all analyzed cathepsins (B, L and S) was significantly reduced compared to uninfected cells (Fig. 4). Our data clearly indicate that during later stages of ECTV infection, together with decreased mRNA expression and protein content, also the cathepsin activity diminishes in cDCs.

### Distribution of selected cathepsins and cystatins in JAWS II and GM-BM cells during ECTV infection

It is well established that both cathepsins B and L are essential enzymes involved in antigen processing [32–34]. Cystatin B acts as inhibitor of these cathepsins and is involved in innate immune response. Deficiency of Cst B leads to down-regulation of IFN regulated genes in murine microglia [21, 35]. Interestingly, it has been shown that cystatin B interacted with several proteins in HIV-1-infected macrophages [36]. Because we observed down-regulation of cathepsins B and L, and cystatin B in ECTV-infected cDCs at gene and protein levels, we investigated whether this change was a genuine reduction in protein translation due to lower mRNA levels or whether the change could have resulted from cytoplasmic relocation of proteins following infection. We visualized protein distribution patterns within infected cells.

**Fig. 5:**
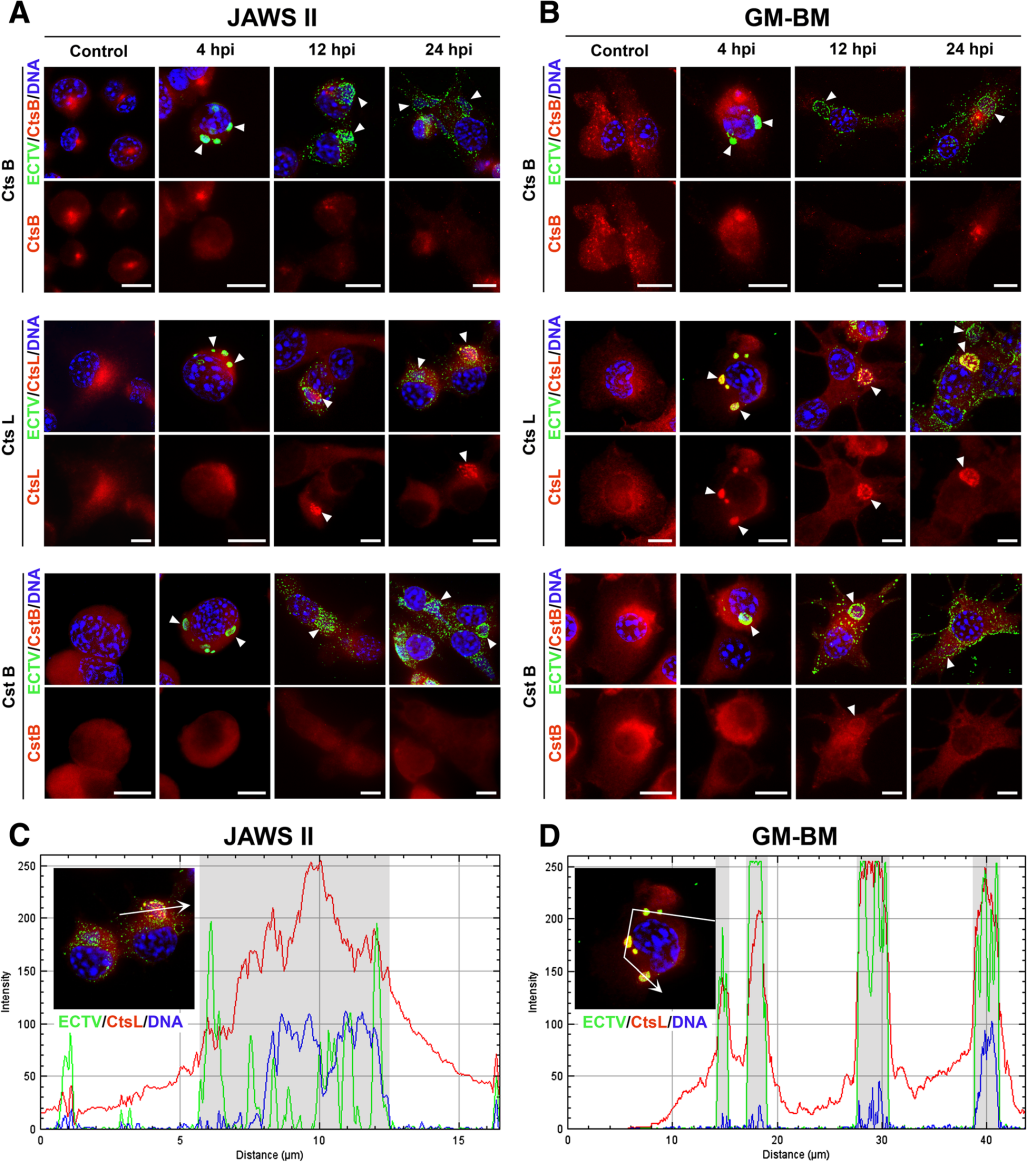
ECTV alters distribution of cathepsins and cystatins in cDCs. Distribution of cathepsins (Cts B and Cts L) and cystatin B (Cst B) in ECTV-infected JAWS II (**a**) and GM-BM (**b**) cells at 4, 12 and 24 hpi. Representative fluorescence microscopy images present intracellular localization of Cts B, Cts L and Cst B (red fluorescence), and pAbs anti-ECTV (green fluorescence). Viral and nuclear DNA were stained with Hoechst 33342. Arrowheads indicate viral factories. Scale bar = 10 μm. The fluorescence intensity of cathepsin L (red), viral antigen (green) and viral DNA (blue) measured along a white line in ECTV-infected JAWS II (**c**) and GM-BM (**d**) cells. White arrows indicate the direction of fluorescence intensity measurement illustrated on graphs. The X-axis represents distance along the line and the Y-axis shows fluorescence intensity values. Grey boxes indicate the location of viral factories

Immunofluorescence analysis revealed that the distribution of cathepsin B was significantly reduced at each time point of infection and no co-localization between cathepsin B and viral factories in JAWS II and GM-BM cells was observed (Fig. 5a and b). Surprisingly, cathepsin L was able to co-localize with viral factories at 4, 12 and 24 hpi both in JAWS II and GM-BM cells (Fig. 5c and d), suggesting that although both are cysteine cathepsins, they may function differently. In the case of cystatin B, immunofluorescence staining showed that cystatin B partially co-localized with viral replication centers especially in GM-BM cells at 4 and 12 hpi (Fig. 5a and b). The data suggests that there is no relocation of cathepsin and cystatins e.g., to the nucleus, protein reduction is highly likely to result from reduced mRNA. The co-localization of cathepsin L and partial co-localization of cystatin B with viral factories may suggest that some ECTV particles transit through intracellular vesicles containing cathepsins and cystatins. However, to verify this hypothesis further investigations are required to determine the direct effect of cathepsins on ECTV viral particles with use cathepsin B-, L- or S-deficient cells together with endosomal and lysosomal markers, including Rab5 for early endosomes, Rab7 for late endosomes and LAMP1 for lysosomes.

### ECTV infection alters receptor-mediated endocytosis and antigen processing in cDCs

**Fig. 6:**
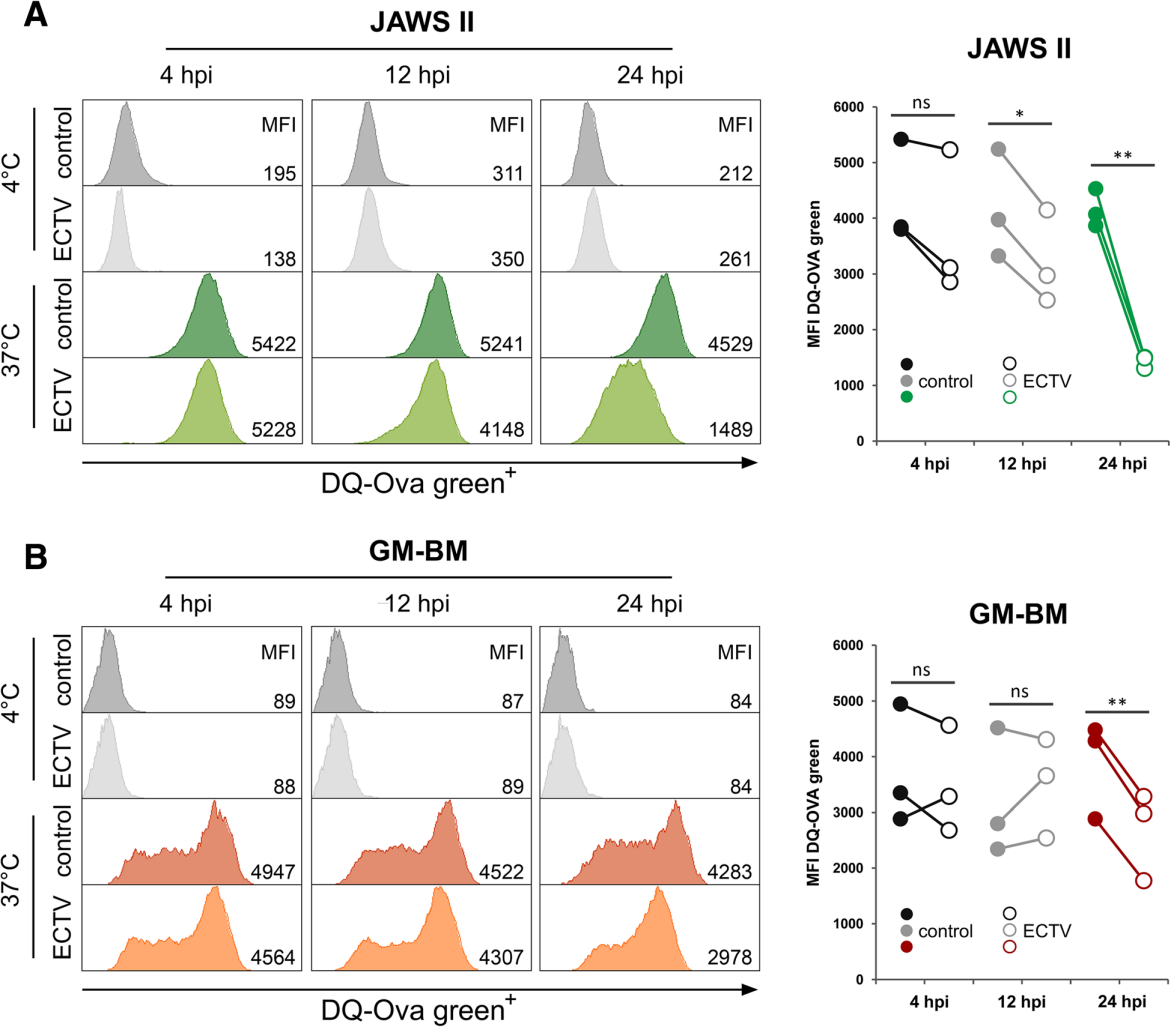
ECTV decreases antigen processing in cDCs at later stages of infection. Antigen uptake and processing in JAWS II (**a**) and GM-BM (b) cells at 4, 12 and 24 hpi with ECTV. Control and infected cells were pulsed with DQ-OVA for 60 min. at 4 °C and 37 °C, and analyzed by flow cytometry. Representative histograms show mean fluorescence intensity (MFI) of DQ-OVA green fluorescence at 4 °C and 37 °C in JAWS II and GM-BM cells. Graphs represent individual data of MFI for DQ-OVA green fluorescence at 37 °C from three independent experiments (*p ≤ 0.05, **p ≤ 0.01)

Cathepsins, including B, L and S, are implicated in antigen processing [37], therefore, we tested the ability of ECTV-infected JAWS II and GM-BM cells to process exogenously-derived antigen. We used a soluble antigen DQ-OVA, conjugated with the pH-insensitive fluorescent dye (BODIPY, FL). After endocytosis through the mannose receptor, DQ-OVA is degraded to bright-green fluorescence fragments by the endo-lysosomal proteases. Uninfected JAWS II and GM-BM cells were able to endocytose and degrade the DQ-OVA, as evidenced by the high MFI value for green fluorescence. Meantime, JAWS II cells infected with ECTV showed significantly (*p* ≤ 0.05) reduced MFI value for green fluorescence at 12 hpi (Fig. 6a), despite the unchanged expression of *Ctsb*, *Cts*l and *Ctss* mRNA and protein expression for cathepsins B and L at this time post infection (Figs. 2, 3). However, at 12 hpi we observed decreased activity of cathepsin L in JAWS II cell lysates (Fig. 4). The reduction in MFI level for green fluorescence in JAWS II cells was highly significant (*p* ≤ 0.01) at 24 hpi. In contrast to JAWS II cells, GM-BM cells did not show reduced MFI values for green fluorescence at 12 hpi, despite significantly (p ≤ 0.05) decreased *Ctsb*, *Ctsl* and *Ctss* expression (Fig. 2b) and reduced level of cathepsin L (Fig. 3b). Therefore, it is possible that other factors than cathepsins may compensate the ability of cDCs to process exogenous antigen during early stages (4 and 12 hpi) of ECTV infection. At 24 hpi GM-BM exhibited significantly (*p* ≤ 0.01) decreased rate of soluble antigen degradation (green fluorescence) compared to the control (Fig. 6b). Taken together, our data indicate that dendritic cells display reduced ability to process a soluble antigen during later stages of infection with ECTV. The reduction of DQ-OVA processing is observed earlier and is more profound in JAWS II than GM-BM cells (at 24 hpi: 2.9-fold and 1.5-fold, respectively). Basing on these findings, we cannot unequivocally state that the enhanced inhibitory effect on DQ-OVA processing observed in both cell types at 24 hpi with ECTV was due to decreased mRNA and protein expression of cathepsins and cystatins (Figs. 2, 3).

### Increased ECTV titer in JAWS II and GM-BM cells after siRNA knock-down of cathepsins

**Fig. 7:**
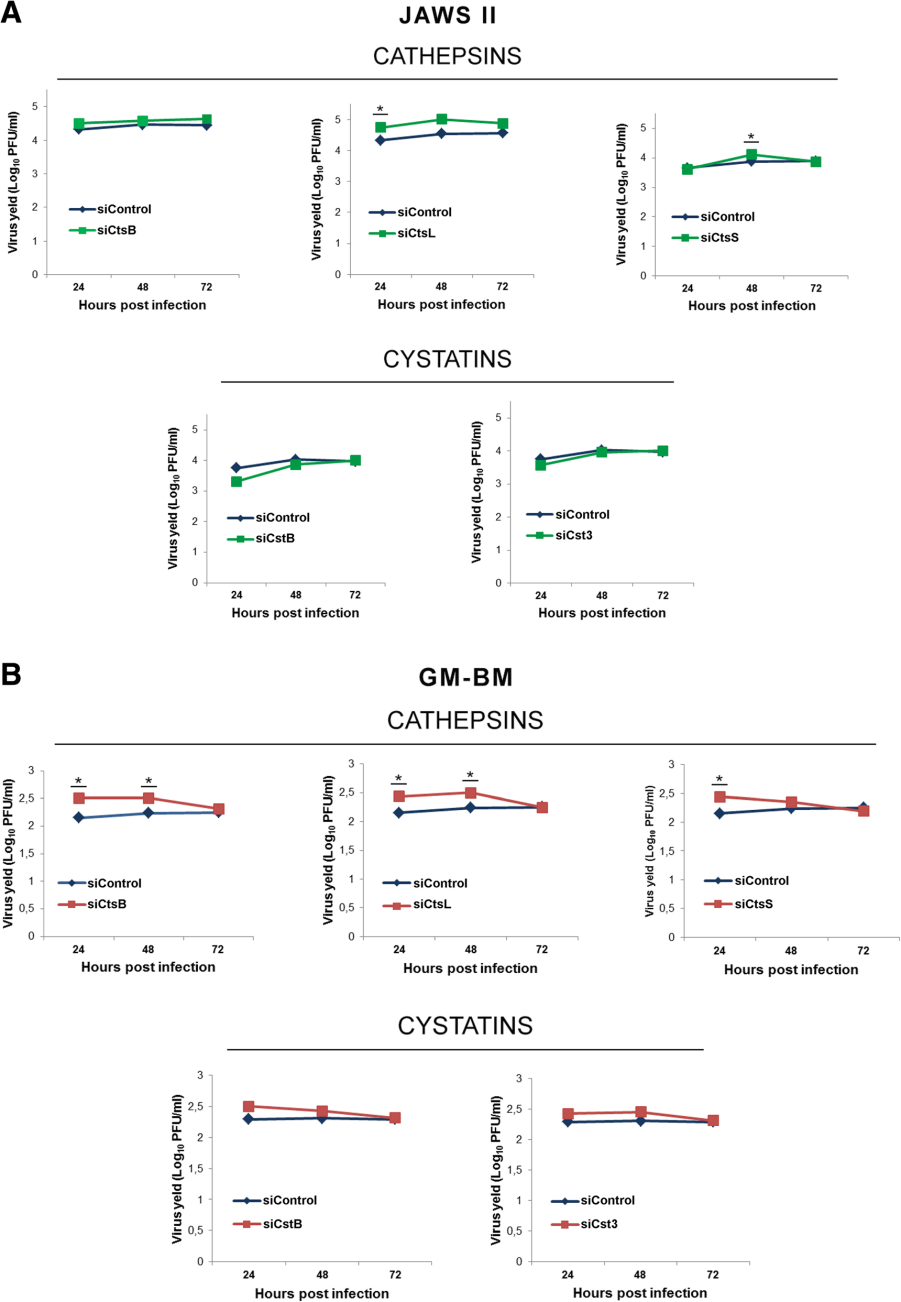
siRNA knock-down of cathepsins increases virus titer in cDCs. Plaque assay determination of ECTV titer in JAWS II (**a**) and GM-BM (**b**) cells treated with control siRNA-A or with siRNA against cathepsin B, L or S and cystatin B or C at 24, 48 and 72 hpi. PFU/ml was calculated from three independent experiments (*p ≤ 0.05)

To investigate the consequence of down-regulation of cathepsins and cystatins at gene and protein level both in JAWS II and GM-BM cells upon infection, we evaluated the direct influence on the replication of ECTV in the near absence of cathepsin and cystatin in these cells. Results show significantly (*p* ≤ 0.05) higher virus titers, indicating efficient ECTV replication in JAWS II cells with knock-down of cathepsin L at 24 hpi, and cathepsin S at 48 hpi compared with control siRNA-A treated cells (Fig. 7a). The gene knock-down of cathepsin B, L and S resulted in significantly (*p* ≤ 0.05) increased viral yield in GM-BM cells at 24 hpi. Moreover, significantly (*p* ≤ 0.05) higher titer of ECTV was observed at 48 hpi in GM-BM cells treated with siRNA against cathepsin B and L compared to control cells without gene knock-down (Fig. 7b). These results, together with markedly decreased expression level of cathepsins at 24 hpi both in JAWS II and GM-BM cells, support our assumption that ECTV suppresses cathepsins as a strategy to efficiently replicate in infected cells. However, ECTV titer after gene knock-down of cystatin B and C in JAWS II and GM-BM cells remained unchanged.

## Discussion

Poxviruses, including ECTV, have evolved numerous mechanisms to evade the host immune response [20]. To further elaborate on the immune evasion strategies engaged by ECTV, we scrutinized the impact of ECTV infection on selected cathepsins (B, L, S) and cystatins (A, B, C) regarding gene and protein expression in murine JAWS II and GM-BM cells. Selection of the cells to analyze was dictated by the fact that dendritic cells are in the forefront of subsequent immune responses ensuing infection. But to be able to attribute any changes to the viral activity only and not to the virus-induced apoptotic process, we first determined the apoptotic rate in murine JAWS II and GM-BM cells. ECTV infection of JAWS II cells at a low MOI (0.5) induces early apoptosis at 24 h following infection, in contrast to GM-BM cells in which the induction of apoptosis was not detected during the entire virus replication cycle. Therefore, the observation at 12 and 24 hpi in both cells types that expression of cathepsins and cystatins was significantly reduced, and degradation of DQ-OVA was altered, indicates that such inhibitory effects were not caused by the apoptotic process and may strictly be related to the immunomodulatory strategies engaged by the virus. Moreover, ECTV itself encodes a variety of proteins to overcome apoptosis [38], including EVM025 that inhibits activity of pro-apoptotic Bak and Bax, and stabilizes mitochondrial membrane integrity [39].

Our results indicated a profound down-regulation of both mRNA and protein expression of cathepsin B, L and S, and cystatins B and C following infection with ECTV both in JAWS II and GM-BM cells, mainly at 24 hpi. It is well documented that poxviruses cause a global shutdown of host mRNA and protein synthesis probably to facilitate their own replication due to deactivation of cellular antiviral response and restriction of competition for the translational machinery [40–42]. This phenomenon would explain why both cathepsins and their negative regulator cystatins are downregulated in ECTV-infected cDCs. Interestingly, we observed that the mRNA expression for cathepsin B, S and cystatin C was primarily reduced in JAWS II cells. Moreover, we observed profound down-regulation of gene expression for cathepsin B, S and cystatin B in GM-BM cells. The level of cathepsin L and S protein was greatly reduced in JAWS II cells, while cathepsin S and cystatin C protein expression was primarily reduced in GM-BM cells. These results are consistent with our previous findings that ECTV infection of peritoneal macrophages isolated from ECTV-infected susceptible BALB/c and resistant C57BL/6 mice have decreased mRNA and protein level of cathepsins B, L and S [24]. We were unable to detect expression of cystatin A in cDCs, however, according to Kopitar-Jerala [21] cystatin A is present only in the follicular dendritic cells (FDCs) that do not internalize, process and present antigens in the context of MHC II. Our findings are in agreement with results obtained by Wang and co-authors [25], where mRNA and protein expression of cathepsins B, D and S was decreased in B cells during VACV infection. However, cathepsin L gene expression and protein level were enhanced in VACV-infected B cells. The authors have suggested that the reduced level of cathepsins may alter antigen processing and probably contributes to the loss of MHC class II function.

Cathepsins have been shown to play essential roles in antigen processing, suggesting that they are involved in adaptive immune responses [43]. For example, studies focused on cathepsin S revealed that this acid-independent lysosomal cysteine protease mediates processing of outer capsid protein σ3 in macrophage-like P388D cells [44]. It is important to note that the role of cysteine cathepsins in APCs, including DCs, is not limited only to antigen processing. Cathepsin B, D and S as well as cystatin C are important enzymes responsible for initiation of removal of the MHC II-associated chaperone invariant chain (li) from MHC II, thus permitting peptide association and intracellular transport of MHC II for display on the APC cell surface. Cathepsin S plays the most important role in this process [45–47]. DCs isolated from cathepsin S-deficient C57BL/6 mice show a dramatically reduced distribution of MHC class II molecules on the cell surface [48]. Other studies also confirmed that cathepsin S is essential for efficient Ii processing, since in the absence of this protease, Ii degradation was markedly diminished in DCs [49] and B cells [50, 51]. Interestingly, cathepsin L-deficient mice also exhibited defective MHC II-associated antigen presentation in cortical thymic epithelial cells (cTECs) as a result of incomplete degradation of Ii. Moreover, in mice with cathepsin L deletion numbers of CD4^+^ T lymphocytes were reduced in the thymus and periphery [52]. Hepatitis C virus (HCV) infection markedly decreased DCs maturation and reduced cathepsin S expression and possibly leading to impaired MHC class II maturation [53]. On the contrary, despite decreased expression of cathepsin B, L and S we observed increased percentage of MHCII^+^ in GM-BM during ECTV infection [30, 31].

Several studies with other viruses have reported that down-regulation of cathepsins impairs endosomal degradation of viral particles and promotes virus survival [10, 11]. For example, cathepsin B inhibition by cystatin C or CA-074Me (synthetic inhibitor) treatment resulted in enhancement of the CD4-independent HIV-1 infection in HeLa and TE671 cells [11]. Harman and colleagues [10] have demonstrated that mRNA and protein expression of cathepsin B, C, S, and Z were profoundly decreased in human monocyte-derived DCs (MDDCs) after 48 h of HIV-1 infection. Moreover, cathepsin L protein level was increased in cells infected with HIV-1. These studies suggest that the reduction of cathepsin activity possibly impairs late endosomal degradation of HIV-1, resulting in uncontrolled replication of virus in DCs. Moreover, authors hypothesized that reduced function of cathepsins may lead to decrease HIV-1 antigen processing and presentation, and therefore diminish HIV-1–specific stimulation of CD4^+^ T lymphocytes to avoid adaptive immune responses [10]. Cathepsin B activity in SAOS-2, HeLa, and TE671 cells was responsible for HIV-1 envelope glycoprotein gp120 degradation in acidic endosomes, contributing to reduced CD4-independent infection [54].

Similar to cathepsin B, down-regulation of cathepsin L also promotes replication of some viruses [55]. Cathepsin L-deficient C57BL/6 mice (*Ctsl*^*−/−*^) infected with influenza A (H1N1) virus were not able to limit viral replication during early stages of infection. Moreover, *Ctsl*^*−/−*^ mice exhibited larger viral loads in lungs and had higher mortality rates than infected *Ctsl*^*+/+*^ mice. These findings suggest a critical role of cathepsin L during influenza A virus infection in mice, as this protease appears to support both innate and adaptive antiviral immune responses [55]. Therefore, in the light of earlier findings by others, our observations suggest that decreased expression of cathepsins and cystatins induced by ECTV in JAWS II and GM-BM cells may serve not only to abrogate antiviral immune response initiated by these cells, but may also provide a more suitable environment for efficient replication in infected cDCs. This statement is supported by our results concerning increased ECTV titer in cDCs after removal of cathepsin B, L or S using siRNA, together with our previous study in which siRNA knock-down of cathepsin B and L in RAW 264.7 macrophages resulted in higher replication of ECTV, compared to infected wild-type cells [24].

ECTV-infected JAWS II and GM-BM cells exhibited decreased cystatin B and C expression both at mRNA and protein level. Čeru and co-workers [56] showed that cystatin B modulates cathepsin L activity in the nucleus, therefore this endogenous inhibitor could play an important role in regulating the proteolytic activity of cathepsin L to protect substrates, such as transcription factors, from proteolytic degradation. Cystatin C regulates cathepsin S activity and Ii processing in DCs [57]. During the process, any reduction in cystatin C-cathepsin S ratio may result in a substantial enhancement in active cathepsin S. Studies on cystatin C derived from human seminal plasma using the dot-blot technique revealed that cystatin C interacted with different HIV-1 proteins, such as gp160, gp120, p31, p24 including HIV protease. Importantly, HIV protease function relies on generating mature functional protein components of an infectious HIV virion. Authors have suggested that if cystatin C was able to inhibit HIV protease activity, it might prevent the normal function of the HIV protease and therefore would potentially prevent viral replication and transmission [58].

Our immunofluorescence analysis revealed evident co-localization of cathepsin L and partial co-localization of cystatin B with viral factories since early stages of ECTV infection in JAWS II and GM-BM cells. Although the distribution of cathepsin B was significantly reduced at each time point of infection, we did not observe co-localization between this protease and viral factories in tested cells. Fiegl and co-workers [59] have observed significant co-localization between *Chlamydia psittaci* and cathepsin D in JAWS II dendritic cell line at 72 hpi. Moreover, authors have indicated that inhibition of cathepsin D or S decreased stimulation of *Chlamydia*-specific CD8^+^ T cells, suggesting a crucial role of these proteases in efficient processing of chlamydial antigens. Moreover, degradation of *Chlamydia* by cathepsins is associated with the generation of bacterial epitopes for antigen presentation in infected JAWS II cells [59]. Other research has demonstrated significant co-localization of *Francisella novicida* with lysosomes in murine *Ctsb*^*−/−*^ bone marrow-derived macrophages (BMDMs), compared with wild type BMDMs at 5 and 12 hpi [60]. Probably, in the absence of cathepsin B the fusion of lysosomes with *F. novicida* was intensified in macrophages. Additionally, it has been speculated that bacteria might require cathepsin B to gain entry to the cytoplasm for further replication. The biological significance of cysteine protease and cystatin co-localization with ECTV replication centers is not known and requires further investigations on the direct effect of endogenous cathepsins on viral particles.

Lysosomal acidification and protein degradation are much lower in DCs than in macrophages and neutrophils, and such reduced degradation allows DCs to maintain antigenic peptides and increased presentation on MHC class I and class II molecules [3]. Because the presentation of antigens by MHC class II molecules is precisely dependent on the function of cathepsins, and our studies revealed down-regulation of cathepsins B, L and mainly S during ECTV infection in JAWS II and GM-BM cells, our last question concerned the ability of these cells to process exogenously-derived antigen. Degradation of DQ-OVA in JAWS II cells was reduced at 12 hpi with ECTV, with the most profound inhibition at 24 hpi. GM-BM cells also displayed altered antigen processing capability only at 24 hpi with ECTV compared to control cells. Taken together, our data indicate that cathepsin down-regulation induced by ECTV is accompanied by decreased ability to process exogenously-derived antigen by cDCs. This may negatively influence the induction of antigen-specific antiviral immune response [31].

Splenic DCs isolated from C57BL/6 mice exhibited decreased MHC class II gene expression and impaired antigen presentation to CD4^+^ T cells on day one after VACV infection. This down-regulation of MHC class II expression in VACV-infected BMDC was possibly caused by E3L – the viral early gene product, but not secreted host factors [61]. Wang and colleagues [25] indicated that mRNA and protein level of MHC class II invariant chain was reduced in B cells from 12 to 14 h of VACV infection. The authors have suggested that decreased cysteine proteases expression, mainly cathepsin S, may be associated with reduced or slow Ii degradation, leading not only to the loss of MHC class II function, but also to efficient transition of virus replication later stages.

## Conclusion

Our results show a profound down-regulation of cathepsins B, L and S, and cystatins B and C in JAWS II and GM-BM cells, together with diminished ability to endocytose and process a soluble antigen by these cDCs during later stages of ECTV infection. Because this highly suggests impaired cellular function, therefore cDCs may not perform efficiently inducing adaptive immune responses. Higher virus titers in cDCs reported in this paper, together with increased viral yield in macrophages after anti-cathepsin siRNA treatment [24], indicate that the virus replicates efficiently during low expression of cathepsin B, L or S. Detailed studies dealing with functions of cathepsins and cystatins in cDCs infected with poxvirus should lead to a better understanding of the poxvirus-host interactions, as this may yield information valuable for the rational design of new vaccines or therapeutic approaches.

## Materials and methods

### Virus

Highly virulent Moscow strain of ECTV (ECTV-MOS, ATCC VR-1374; Manassas, VA, USA) was used in all experiments. ECTV was propagated and titrated by plaque formation assay (PFU/ml) in Vero cells (ATCC CCL-81). Viral stocks were stored in aliquots at − 70 °C until used.

### Animals

C57BL/6 male mice (8–12 weeks old) were purchased from the animal facility at Maria Skłodowska-Curie Memorial Cancer Centre and Institute of Oncology in Warsaw, Poland. Animals were allowed to acclimate at animal facilities at the Faculty of Veterinary Medicine (registration no. 14313537) for 1 week before experimental procedures. Mice were given *ad libitum* access to food and water. The experimental procedures were approved by the 3^rd^ Ethical Committee for Animal Experimentation at Warsaw University of Life Sciences – SGGW (permission no. 34/2012) and were conducted according to the institutional Guidelines for Care and Use of Laboratory Animals. The total number of animals used for GM-BM generation was 12. Mice were sacrificed by cervical dislocation, and femurs and tibiae were removed for preparation of GM-BM cells. This work adheres to ARRIVE guidelines (Additional file 1: Figure S1).

### Generation and infection of GM-BM cells

GM-BM cells were obtained, as previously described [31]. Enrichment and evaluation of cell surface marker expression on GM-BM was performed, as previously reported [30, 31]. Cells were purified by MACS separation and the purity and phenotype of CD11c^+^ cells were assessed by flow cytometry, as previously described [30, 31]. CD11c^+^ cells were resuspended in complete RPMI-1640 medium (control cells) or exposed to live-ECTV at MOI of 0.5. Virus adsorption was carried out at 37 °C in a humidified 5% CO_2_ atmosphere for 60 min. Control and ECTV-infected cells were cultured for 4, 12 and 24 h.

### Cultivation and infection of JAWS II cells

Immature dendritic cells JAWS II (ATCC CRL-11904) were maintained in alpha-minimum essential medium (MEM) with ribonucleosides, deoxyribonucleosides (Corning, Corning, NY, USA) supplemented with 20% heat-inactivated fetal bovine serum (FBS, HyClone, Logan, UT, USA), 1% antibiotic solution containing 100 U/ml penicillin and 100 μg/ml streptomycin (HyClone), 4 mM L-glutamine (HyClone) and 5 ng/ml rmGM-CSF (R&D Systems, Minneapolis, MN, USA). JAWS II cells were infected with ECTV in the same manner as GM-BM at MOI of 0.5.

### Apoptosis measurement

Control (uninfected) and ECTV-infected JAWS II and GM-BM cells were collected at 4, 12 and/or 24 hpi and stained with Annexin V-FITC and propidium iodide (PI) using the FITC Annexin V Apoptosis Detection Kit I (BD Biosciences, San Jose, CA, USA), according to the manufacturer’s instructions. Annexin V-FITC and PI negative cells were assessed as viable cells, Annexin V-FITC positive and PI negative as early apoptotic cells, and Annexin V-FITC and PI positive as late apoptotic cells. Cells were analyzed immediately using BD LSR Fortessa flow cytometer (BD Biosciences, USA).

### Real-time quantitative PCR

Total RNA was isolated from 7 × 10^5^ control and ECTV-infected JAWS II or GM-BM cells at 4, 12 and 24 hpi using the Qiagen RNAeasy Mini Kit (Qiagen, Inc., Valencia, CA, USA), as recommended by the manufacturer, and used to assess mRNA expression for selected cathepsin genes (B (*Ctsb*), L (*Ctsl*), S (*Ctss*) and cystatin genes: A (*Csta*), B, (*Cstb*) and C (*Cst3*)). Genomic DNA was removed during RNA purification using RNase-Free DNase on column digestion (Qiagen). RNA concentration was measured in the NanoDrop 1000 spectrophotometer (Thermo Fisher Scientific, Waltham, MA, USA). RNA quality was assessed in 2100 Bioanalyzer using Agilent 6000 Nano Kit (Agilent Technologies, Santa Clara, CA, USA). RNA integrity number (RIN) was 10. Next, reverse transcription was done using the RT^2^First Strand Kit (Qiagen), according to the manufacturer’s protocol in gradient thermocycler SensoQuest Labcycler (Göttingen, Germany). cDNA was then mixed with TaqMan Universal Master Mix II no UNG (Applied Biosystems; Thermo Fisher Scientific), TaqMan Gene Expression Assays (Table 1) (Applied Biosystems) according to the manufacturer’s protocol. Amplification was conducted in 7900HT Fast Real-Time PCR System (Thermo Fisher Scientific) at 95 °C for 10 min, 45 cycles of 95 °C for 15 s and 60 °C for 1 min. Average threshold cycle (C_T_) values from PCR reactions were normalized against *Cdkn1a* and shown as 2^(−ΔΔCT) were ΔC_T_ = C_T Gene_ − C_T Cdkn1a._

**Table 1:** Assay ID and amplicon length of Taqman Gene Expression Assays used

Gene	Assay ID	Amplicon length
*Ctsb*	Mm01310506_m1	94
*Ctsl*	Mm00515597_m1	87
*Ctss*	Mm01255859_m1	75
*Csta1*	Mm01344699_g1	115
*Cstb*	Mm00432769_m1	134
*Cst3*	Mm00438347_m1	77
*Hsp90ab1*	Mm00833431_g1	167

### Western blot analysis

Western blot analysis was performed to determine protein levels of the selected cathepsins and cystatins in GM-BM and JAWS II cells. Control and ECTV-infected cells were harvested after 4, 8, 12 and/or 24 hpi. Next, the cells were lysed using RIPA buffer (Thermo Fisher Scientific) supplemented with 1% protease inhibitor cocktail (Thermo Fisher Scientific). Protein concentration in lysates was measured via bicinchoninic acid (BCA) assay (Thermo Fisher Scientific) and spectrophotometry on Epoch BioTek spectrophotometer (BioTek Instruments, Inc., Winooski, VT, USA). Next, 20 μg of protein content in lysates were electrophoresed on 12% polyacrylamide Bis-Tris Plus gel with MES running buffer (both from Thermo Fisher Scientific) and transferred to polyvinylidene fluoride (PVDF) membranes. PVDF membranes were blocked in 5% non-fat dry milk with 0.1% Tween 20-PBS solution and incubated with primary antibodies overnight at 4 °C as follows: cathepsin B (Ctsb) – 1:500 (Abcam, Cambridge, UK), cathepsin L (Ctsl) – 1:400 (Abcam), cathepsin S (Ctss) – 1:400 (Santa Cruz Biotechnology, Dallas, TX, USA), cystatin B (Cstb) – 1:1000 (Thermo Fischer Scientific) and cystatin C (Cst3) – 1:10000 (Abcam). Protein bands were detected with secondary anti-goat or anti-mouse antibodies conjugated to horseradish peroxidase (HRP, 1:5000) purchased from Santa Cruz Biotechnology. Glyceraldehyde 3-phosphate dehydrogenase (GAPDH, Thermo Fisher Scientific) was used as a protein loading control. Chemiluminescence was developed with the SuperSignal West Pico Chemiluminescent substrate (Thermo Fischer Scientific). The protein bands were visualized by autoradiography. Densitometry analysis was performed using ImageJ software (NIH, Bethesda, MD, USA).

### Measurement of cathepsin activity

Activity of cathepsin B, L and S was assessed using the Cathepsin B, L or S Activity Assay Kit, respectively, (Abcam, Cambridge, UK), according to the manufacturer’s instructions. Briefly, 2.5 × 10^6^ uninfected or ECTV-infected JAWS II cells at 4, 12 and 24 hpi were lysed using 50 μl of Cell Lysis Buffer and centrifuged at 14000 g for 5 min at 4 °C. Next, 50 μl of lysate from each sample was transferred into separate wells of 96-well microplate for fluorescence-based assays (Thermo Fisher Scientific, Waltham, MA, USA). Then, 50 μl of Reaction Buffer and 2 μl of appropriate Substrate (Ac-RR-AFC for cathepsin B, Ac-FR-AFC for cathepsin L and Ac-VVR-AFC for cathepsin S) were added to each well containing 50 μl of sample. Additionally, 1 μl of dithiothreitol (DTT) was added to each cathepsin L sample. Negative control samples, containing cathepsin B, L or S Inhibitors were also included in the assay. The plate was incubated at 37 °C for 2 h protected from light and fluorescence was measured at Ex/Em = 400/505 nm using a microplate reader Infinite 2000 Pro (Tecan, Männedorf, Switzerland).

### Immunofluorescent staining and microscopy analysis

JAWS II and GM-BM cells were seeded on coverslips placed in a 24-well plate at a density of 1.5 × 10^5^ cells per well. Cells were left uninfected or were treated with ECTV for 60 min. at 37 °C. After 4, 12 and 24 h, cells were fixed with 4% paraformaldehyde (PFA, Sigma-Aldrich, St Louis, MO, USA) and permeabilized with 0.5% Triton X-100 (Sigma-Aldrich) in PBS. Next, JAWS II and GM-BM cells were blocked with 3% bovine serum albumin (BSA, Sigma-Aldrich) in 0.1% Triton X-100 and incubated for 45 min. with anti-cathepsin B, anti-cathepsin L (both from Abcam, Cambridge, MA, USA) and anti-cystatin B (Thermo Fisher Scientific) primary antibodies. After washing with 0.1% Triton X-100 in PBS, cells were incubated with secondary anti-mouse or anti-rabbit antibodies conjugated with rhodamine Red-X (Jackson ImmunoResearch Laboratories, Inc., West Grove, PA, USA) diluted in blocking solution for 1 h. ECTV antigens were stained with FITC-conjugated polyclonal antibodies for 1 h. Viral and nuclear DNA was stained with Hoechst 33342 (Sigma-Aldrich) solution for 10 min. in the dark. Slides were mounted in ProLong Gold Antifade Reagent (Life Technologies). Images were captured using Olympus BX60 fluorescence microscope and analyzed with Cell^F software (Soft Imaging System, Olympus, Tokyo, Japan) and ImageJ (NIH, Bathesda, MD, USA).

### Detection of antigen uptake and processing

Endocytosis and processing of ovalbumin (OVA) were analyzed using DQ-OVA (OVA labeled with boron-dipyrromethene, BODIPY FL dye; Molecular Probes, Eugene, OR, USA). The fluorescence of DQ-OVA is self-quenching until the OVA is taken up through the mannose receptor-mediated endocytosis and degraded by endosomal/lysosomal proteases. Control and ECTV-infected JAWS II and GM-BM cells at 4, 12 and 24 hpi were incubated with 30 μg/ml DQ-OVA at 37 °C or at 4 °C for 60 min. Cells were then washed three times with ice-cold PBS cells and analyzed in BD LSR Fortessa flow cytometer.

### siRNA knock-down of cathepsins and cystatins in JAWS II and GM-BM cells

JAWS II and GM-BM cells were grown in MEM with ribonucleosides, deoxyribonucleosides supplemented with 20% heat-inactivated FBS, 4 mM L-glutamine and 5 ng/ml rmGM-CSF and in RPMI-1640 medium containing 10% heat-inactivated FBS, 50 mM 2-mercaptoethanol and 20 ng/ml rmGM-CSF, respectively, and seeded at 1 × 10^5^ per well in 24-well plates. After 24 h cells were treated with siRNA against cathepsin B, L or S and cystatin B or C. Appropriate amounts of siRNA duplex (Santa Cruz Biotehnology) and siRNA Transfection Reagent (Santa Cruz Biotehnology) diluted with enough Plasmid Transfection Medium (Santa Cruz Biotehnology) was determined empirically. The cells were incubated 7 h under normal conditions (37 °C, 5% CO_2_). After that 250ul of normal growth medium containing 20% FBS was added without removing the transfection mixture. 24 h post-transfection the medium was aspirated and replaced with fresh normal growth medium. Cells were incubated for 48 or 72 h after transfection at 37 °C in a CO_2_. Control cells remained untreated and negative control cells were transfected with siRNA-A that does not lead to genes knock-down. Gene silencing was confirmed by measuring protein content in lysates by Western blot (Additional file 2: Figure S2). GAPDH, was used as a protein loading control.

### Plaque assay

To investigate the impact of cathepsin B, L or S, and cystatin B or C knockdown on the replication of ECTV, JAWS II and GM-BM cells after siRNA treatment were infected with ECTV at MOI = 0.5. Supernatants and cells were collected after 24, 48 and 72 h and clarified before use in a plaque assay to determine the viral titers. Vero cells cultured on 24-well plates were inoculated with 10-fold serial dilutions of supernatants from siRNA treated ECTV infected cells and control siRNA-A treated cells. After 5 days, plaques were fixed in 4% PFA and counted under Olympus IX71 inverted microscope.

### Statistical evaluation

All results were presented as the arithmetic means ± standard deviation (SD) of at least three independent experiments. The significance of statistical differences was evaluated for the normal distribution of data with the use two-dependent (paired) or two-independent (unpaired) Student’s t-tests (STATISTICA 6.0 software, StatSoft Inc., Tulsa, OK, USA). Significant differences were shown with *p ≤ 0.05, **p ≤ 0.01 or ***p ≤ 0.001.

## Additional files

Additional file 1: **Figure S1** The ARRIVE guidelines checklist. (PDF 389 kb)

Additional file 2: **Figure S2** Confirmation of gene knockdown of cathepsin B, L or S, and cystatin B or C in JAWS II (A) and GM-BM (B) cells. The level of each protein was normalized to GAPDH. Western blots of control non treated cells, control siRNA-A treated cells and cells treated against CtsB, CtsL, CtsS, CstB and Cst3 at 48 and 72 h of siRNA treatment in JAWS II and GM-BM cells. (TIF 2400 kb)

## Abbreviations

APC: Antigen presenting cell
BCA: Bicinchoninic acid
BMDM: Bone marrow-derived macrophage
cDC: Conventional dendritic cell
cTEC: Cortical thymic epithelial cell
DC: Dendritic cell
EBOV: Ebola virus
ECTV: Ectromelia virus
FBS: Fetal bovine serum
FDC: Follicular dendritic cell
GAPDH: Glyceraldehyde 3-phosphate dehydrogenase
GM-BM: GM-CSF-derived bone marrow cell
HCV: Hepatitis C virus
HIV: Human immunodeficiency virus
hpi: Hour post infection
HRP: Horseradish peroxidase
IFN: Interferon
li: Invariant chain
MDDC: Human monocyte-derived dendritic cell
MEM: Alpha-minimum essential medium
MHC: Major histocompatibility complex
MOI: Multiplicity of infection
NF-κB: Nuclear factor κB
PI: Propidium iodide
PVDF: Polyvinylidene fluoride
RIN: RNA integrity number
STAT: Signal transducer and activator of transcription
TLR: Toll-like receptor
VACV: Vaccinia virus
VARV: Variola virus
