# Supplementary figures and images for: Ectromelia virus suppresses expression of cathepsins and cystatins in conventional dendritic cells to efficiently execute the replication process
Source: BMC Microbiol. 2019 May 10;19:92. doi: 10.1186/s12866-019-1471-1 (PMC6509786; doi:10.1186/s12866-019-1471-1)

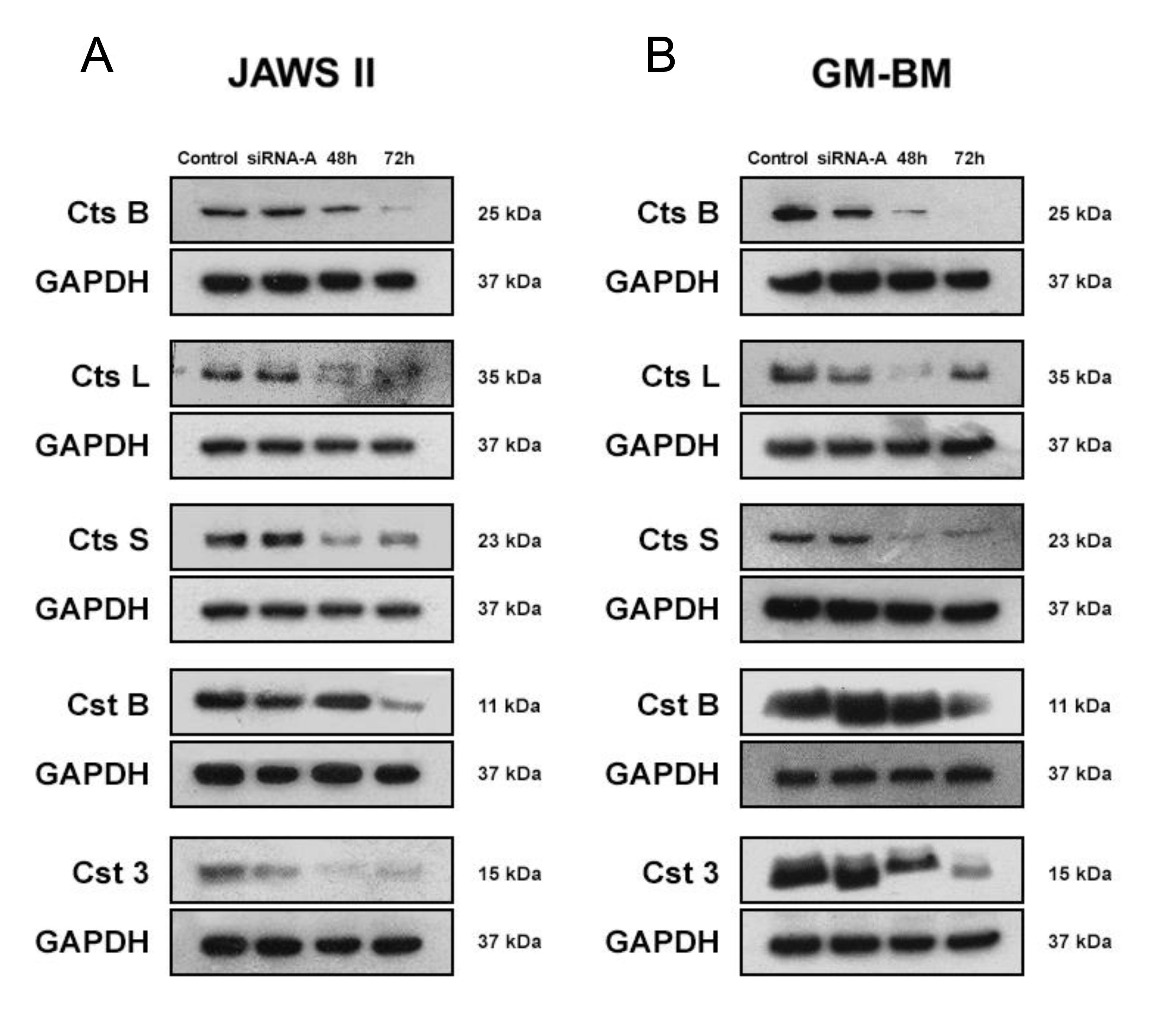

Supplement: Supplementary file 2 — Figure S2 Confirmation of gene knockdown of cathepsin B, L or S, and cystatin B or C in JAWS II (A) and GM-BM (B) cells. The level of each protein was normalized to GAPDH. Western blots of control non treated cells, control siRNA-A treated cells and cells treated against CtsB, CtsL, CtsS, CstB and Cst3 at 48 and 72 h of siRNA treatment in JAWS II and GM-BM cells. (TIF 2400 kb) [file 12866_2019_1471_MOESM2_ESM.tif]
